# Perilipin polymorphisms are risk factors for the development of obesity in adolescents? A case-control study

**DOI:** 10.1186/s12944-017-0440-7

**Published:** 2017-03-09

**Authors:** Yavuz Tokgöz, Ishak Abdurrahman Işık, Soheil Akbari, Tuncay Kume, Oya Sayın, Esra Erdal, Nur Arslan

**Affiliations:** 1 Department of Pediatric Gastroenterology, Hepatology and Nutrition, Dokuz Eylul University Faculty of Medicine, 35330 Inciraltı-Izmir, Turkey; 2 Department of Medical Biology, Dokuz Eylul University, Faculty of Medicine, Izmir, Turkey; 3 Department of Biochemistry, Dokuz Eylul University, Faculty of Medicine, Izmir, Turkey; 4 Research Laboratory, Dokuz Eylul University, Faculty of Medicine, Izmir, Turkey; 5 Department of Pediatric Metabolism and Nutrition, Dokuz Eylul University, Faculty of Medicine, Izmir, Turkey

**Keywords:** Adolescent, Perilipin polimorphisms, Obesity, Adipokines

## Abstract

**Background:**

The variations in perilipin gene (PLIN) were previously associated with obesity. We examined the association of polymorphisms at the *PLIN* locus in adolescents with obesity and their connection with serum adipokines.

**Methods:**

A total of 308 children (206 obese, 66.8% and 102 healthy control, 33.2%) between the ages of 10-18 years were included into the study. *PLIN* gene analysis [PLIN 1, PLIN 4, PLIN 6, PLIN 5’UTR-1234 C > G and PLIN 10171 A/T] were studied by Real Time-PCR. Serum leptin, adiponectin, resistin and ghrelin levels were studied by ELISA method in both groups and their link with perilipin polymorphisms were analyzed.

**Results:**

Serum leptin level was found significantly high in obese adolescents. Other adipokine levels were similar in both groups. The incidence of PLIN 1, PLIN 4, PLIN 5’UTR-1234 C > G and PLIN 10171 A/T minor and major alleles was similar in both groups. PLIN 6 T/T allele was determined significantly high in obese adolescents compared to that of control group. No correlation was detected between perilipin polymorphism and serum levels of adipokines.

**Conclusion:**

The PLIN 6 polymorphism of the perilipin gene may influence the risk of the obesity during adolescence.

**Trial registration:**

Retrospectively registered.

## Background

Until recently, white adipose tissue has been thought to be a simple fat storage. Nevertheless, after adipose tissue was understood to have a role in the metabolism sex steroids and leptin was discovered, this conventional thought has changed completely. The idea that white adipose tissue is not a simple fat storage and it can have a part in the energy balance of the body has formed a ground. Thereafter, over 50 types of cytokines and other molecules released from this tissue have been described and commenced to be referred to as adipokine. These adipokines are linked with endocrine, paracrine and other mechanisms and they play a role in many physiological and pathological events. These proteins play a role in the procurement of energy balance in short or long periods. Adiponectin, leptin, resistin and ghrelin involved in this group are proteins possessing a crucial role in the lipid metabolism [1–3].

The other important component in the regulation of fat tissue metabolism is perilipins. Perilipins are phosphorylated proteins present on the surface of lipid droplets in the adipocytes, steroid producing cells, liver, heart and muscle cells [4, 5]. Perilipin gene is on the chromosome 15q26 and there are also connection fields for diabetes, hyperlipidemia and obesity in this very area. At least 11 different “single nucleotide polymorphisms” (SNP’s) have been defined in human perilipin gene. The link between SNP’s and clinical situations including various races, sex, obesity in the age groups, glucose intolerance, insulin resistance, metabolic syndrome was searched. Some connections between PLİN1 SNP’s in particular and obesity were identified. PLIN SNP’s were rs2289487, rs894160, rs1052700 and rs2304795 whose apparent relation was determined with obesity and on which most studies have been performed. PLIN1 SNPs rs2289487 and rs894160 were reported to be protective against lipoidosis. PLIN1 rs1052700 and rs2304795 were linked with increased obesity risk [6].

There are a limited number of studies analyzing the perilipin polymorphisms and their connection with adipokines in adolescents with obesity. We analyzed perilipin polymorphisms mostly encountered in obese adolescents and their relation with adipokine levels.

## Methods

### Study population

Patients (between 2011-2012, aged between 10 to 18 years) referred to Dokuz Eylül University Department of Pediatric Gastroenterology, Hepatology and Nutrition with the complaint of excess weight and obesity diagnosed according to the anthropometric measurements formed the obese group. Patients with genetic diseases (Prader-Willi syndrome, Down Syndrome) and chronic diseases (Cushing Syndrome, growth hormone deficiency, diabetes mellitus and hypothyroidism) and patients on medications (e.g. corticosteroids, anti-diabetics) were excluded.

A complete physical examination and anthropometric measurements were applied to all patients. Height was measured in stocking feet to the nearest 0.5 cm using a stadiometer. Body weight was measured by using calibrated scales in light clothing to the nearest 0.1 of a kg. Height and body weight measurements were taken twice and the mean of two readings was calculated. The body mass index (BMI) was calculated as weight (in kilograms) divided by height (in meters squared). Patients whose lean body mass index was higher than 95^th^ percentile were diagnosed as obese [7].

The control group consisted of asymptomatic healthy children and adolescents who were admitted to the Healthy Child Outpatient Clinic of our hospital for medical screening (screening for hepatitis, thyroid functions, lipid profile, celiac disease or anemia etc). All of these screening test results were normal. Anthropometric values and abdominal ultrasound of the controls were in the normal ranges. Serum glucose levels, lipid profiles and liver function tests of the control group were performed and found within the normal ranges.

### Biochemical analysis

Venous blood samples were centrifugated at 3000 *g* at 4 °C for 15 min. Serums were frozen at -20 °C until enzyme-linked immunosorbent assay (ELISA) analysis. Leptin serum concentration was measured by using human leptin ELISA kit (Catalog No. EK0437,Boster Biological Technology Co., Ltd.). The samples were read on a Synergy HT, Multi-Detection Microplate Reader, BIO-TEK plate reader by measuring the absorbance at a wavelength of 450 nm. The ELISAs were performed according to the instructions of the manufacturerand the intra and interassay coefficients of variation were < 10%. All assays were conducted twice in the same occasion and the average value obtained, and conducted within the same laboratory under the same conditions.

### Statistical analysis

Statistical analysis was performed by SPSS Software 15.0. Data were shown as mean ± standard deviation and odds ratio, 95% confidence interval. The χ2 test was used to assess whether the genotypes were in Hardy–Weinberg equilibrium and to compare the genotype and allele frequencies between case and control subjects. Differences in mean values between groups were analyzed with student-t test or with Mann-Whitney U-test, when sample size is smaller than 30. To determine the correlation between two groups, Pearson correlation analysis was performed. All *p* values are two-tailed and group differences or correlations with *p* <0.05 were accepted as significant.

## Results

### Antropometric and clinical data

A total of 308 children and adolescents were included. 206 formed the obese group and 102 healthy children accounted for the control group. There were no statistical differences between obese and control groups with regard to age (obese group: 12.7 ± 2.14 years, control group: 13.1 ± 2.55 years, *p* = 0.51) and sex (male *n* = 157, female *n* = 151, *p* = 0.70). Body weight, BMI and BMI percentile of adolescents in obese group were significantly higher than those of control group. Serum total cholesterol, triglyceride, fasting insulin and HOMA-IR index in obese adolescents were found significantly higher those of control group (*p* < 0.05). Systolic and diastolic blood pressures in both groups were similar (*p* > 0.05) (Table 1).

**Table 1:** Clinical characteristics of obese and nonobese adolescents

	Obese (*n* = 206)	Control (*n* = 102)	*p*
Age (year)	12.7 ± 2.1	13.1 ± 2.55	0.51
Sex (n)			
Male	113 (54.9%)	44 (43.1%)	0.69
Female	93 (45.1%)	58 (56.9%)	
Weight (kg)	71.8 ± 19.1	51.3 ± 11.2	*0.000*
Height (cm)	156.6 ± 11.1	159.9 ± 12.8	0.12
BMI (kg/m^2^)	28.9 ± 5.2	19.2 ± 2.0	*0.000*
BMI percentile (kg/m^2^)	96.3 ± 3.4	56.1 ± 23.9	*0.000*
BP systolic (mm Hg)	113 ± 16	108 ± 10	0.25
BP diastolic (mm Hg)	72 ± 12	70 ± 10	0.33
Glucose (mg/dl)	86.8 ± 8.2	82.5 ± 7.7	0.41
TC (mg/dl)	172.9 ± 33.6	112.2 ± 29.6	*0.001*
LDL (mg/dl)	104.5 ± 30.1	85.3 ± 22.5	*0.001*
HDL (mg/dl)	44.5 ± 10.2	42.1 ± 10.5	0.50
Insulin (μU/ml)	11.8 ± 10.4	5.7 ± 4.4	*0.001*
HOMA-IR	2.54 ± 2.4	1.2 ± 0.9	*0.000*

### Serum adipokines

Serum leptin level was established significantly high in adolescents with obesity compared to that of control group. Ghrelin, adiponectin and resistin levels, however, were identified at the similar levels (Table 2).

**Table 2:** Serum adipokines levels of obese adolescents and control group

	Obese	Control	*p*
Adiponectin (ng/mL)	5.6 ± 2,9	5.0 ± 1.9	*0.07*
Leptin (pg/mL)	11.3 ± 2.3	7.5 ± 3.9	*0.0001*
Resistin (pg/mL)	17.4 ± 6.9	19.5 ± 6.1	*0.08*
Ghrelin (ng/mL)	2.46 ± 0.70	2.50 ± 0.69	*0.56*

### PLIN polymorphisms

In control group, PLIN polymorphisms (PLIN1, PLIN4, PLIN6, rs4578621, rs8179043) and allele incidence were found between 0,21 and 0,30 and genotype distribution did not significantly deviate from Hardy–Weinberg equilibrium (*P* > 0.05). We first examined the associations between PLIN genotypes and obese and nonobese adolescents. Frequency of homozygote rs1052700 (T/T) allele was significantly higher in obese group compared to that of control group (*p* = 0.02, OR = 1.96 [1.11-3.45]). The frequency of other *PLIN* alleles were similar in two groups (Table 3).

**Table 3:** *PLIN* SNP’s in obese and healthy children

		Obese	Control	*p*	OR
		*n* = 206, (%)	*n* = 102,%		
rs2289487 (*PLIN* 1)	G/G	139 (67.5)	69, (67.7)	0.78	0.90 (0.53-1.54)
	A/A+ G/A	67 (32.5)	33, (32.3)		
rs894160 (*PLIN* 4)	G/G	88, (42.7)	54, (52.9)	0.84	0,64 (0.39-1.05)
	A/A+ G/A	118, (47,3)	48, (47.1)		
rs1052700 (*PLIN* 6)	T/T	165, (80.1)	68, (66.7)	*0.02*	1.96 (1.11-3,45)
	A/A+ T/A	41, (19.9)	34, (33.3)		
rs4578621	G/G	102, (49.5)	53, (52.0)	0,71	0.90 (0.56-1.46)
	C/C+ G/C	104, (50.5)	49, (48.0)		
rs8179043	G/G	116, (56.4)	48, (47.0)	0.16	1.43 (0.87-2.34)
	A/A+ G/A	90, (43.6)	54, (53.0)		

### The relationship of PLIN polymorphisms with adipokines

We also assessed the relationship between PLIN genotypes and serum adipokines. We found that no correlation has been detected between minor and major alleles of rs894160, rs1052700, rs2289487, rs4578621, rs8179043 and mean serum levels of adiponectin, leptin, resistin and grelin in our study (Table 4).

**Table 4:** Averages of serum adipokines in *PLIN* SNP’s

		Leptin	*p*	Adiponectin	*p*	Resistin	*p*	Ghrelin	*p*
		(pg/mL)		(ng/mL)		(pg/mL)		(ng/mL)	
rs2289487 (*PLIN* 1)	G/G	9,8 ± 3,7	*0.17*	5,7 ± 3,2	*0,66*	18,1 ± 6,9	*0,9*	2,5 ± 0,67	*0,2*
	A/A+ G/A	10,4 ± 3,1		5,5 ± 2,9		18,1 ± 6,6		2,3 ± 0,74	
rs894160 (*PLIN* 4)	G/G	10,1 ± 3,2	*0,72*	5,6 ± 2,8	*0,95*	18,6 ± 5,9	*0,34*	2,5 ± 0,67	*0,65*
	A/A+ G/A	9,9 ± 3,7		5,7 ± 3,3		17,7 ± 7,4		2,4 ± 0,72	
rs1052700 (*PLIN* 6)	T/T	10,1 ± 3,4	*0,34*	5,7 ± 3,0	*0,97*	18,2 ± 6,9	*0,78*	2,4 ± 0,72	*0,90*
	A/A+ T/A	9,6 ± 3,8		5,7 ± 3,2		17,9 ± 6,3		2,4 ± 0,09	
rs4578621	G/G	10,0 ± 3,5	*0,94*	5,5 ± 3,0	*0,64*	18,6 ± 6,9	*0,37*	2,4 ± 0,71	*0,88*
	C/C+ G/C	10,0 ± 3,4		5,7 ± 3,0		17,8 ± 6,4		2,4 ± 0,69	
rs8179043	G/G	10,2 ± 3,3	*0,20*	5,7 ± 2,9	*0,91*	17,8 ± 7,4	*0,38*	2,4 ± 0,71	*0,45*
	A/A+ G/A	9,6 ± 3,8		5,7 ± 3,2		18,5 ± 5,7		2,5 ± 0,69	

## Discussion

Perilipins are phosphorylated proteins present on the surface of lipid droplets in the adipocytes, steroid producing cells, liver, heart and muscle cells. These proteins have a central role in mobilization and storage of the triglycerides in the adipose tissue [8]. In the fasting state, catecholamines bind their cell surface receptors, initiate signals that activate cAMP-dependent protein kinase (PKA); PKA stimulate the phosphorylation of perilipin A and lastly, activated perilipin A facilitates lipolysis in the adipocytes. In basal conditions, however, (as long as there is no hunger) perilipins facilitate fat deposition by preventing lipolysis [8, 9].

A number of studies investigating the effect of mutation of genes producing perilipins on lipid metabolism have increased recently. The preventive role of PLIN gene mutation in high fat diet associated with obesity had been demonstrated in rodents [9–11]. About 60-70% decrease in body fat stores, normal body weight in spite of high food consumption, increase in muscle mass and metabolic rate and maximum lipolysis in basal metabolic status had been demonstrated in PLIN-/- phenotype [12].

In human studies, common variations of in the PLIN gene have been linked with various risk factors such as diabetes, obesity, weight gain, insulin resistance and hypertension [6]. PLIN 1 6209 T-C (rs2289487) and PLIN 4 11482 G-A (rs894160) are most frequently investigated perilipin SNP’s. Qi and colleagues demonstrated that PLIN 1 and PLIN 4 gene variations were associated with sex and minor alleles of PLIN 1 and PLIN 4 were also related to low BMI in Spanish women population [13]. The presence of PLIN 4 minor allele had been shown to cause decrement in perilipin levels and increment in lypolitic activity, stated by Mottagui-Tabar and colleagues [14]. Perez-Martinez and colleagues had demonstrated that the presence of minor C and A alleles in PLIN 1 and PLIN 4 resulted in low postprandial and atherosclerotic response [15]. Sone and colleagues had shown that PLIN 4 polymorphism was associated with body weight in middle aged Japanese males [16]. The association of PLIN 4 minor allele with metabolic syndrome had been demonstrated in a study conducted with 234 obese children and adolescents [17]. We could not show any association between PLIN 1 and 4 polymorphisms and obesity. Similar results had been shown in some other studies. In a study performed in France, PLIN 4 had been indicated not to be linked to anthropometric measurements, plasma leptin, glucose and insulin concentrations [18]. Similar results had been obtained in China. They had investigated the association between PLIN 1,4,5,6 and obesity and they could not show any correlation. However, the ethnic factors were also found to be important in developing obesity in case of different PLIN gene polymorphisms [19].

PLIN 6 14995 A/T (rs1052700) is another SNP investigated to be associated with obesity in a few studies. Qi and colleagues demonstrated that PLIN 5 and 6 were associated with high body fat mass and waist circumference in women. A-T and G-T alleles were connected to obesity rather than A-A allele [13]. In another study performed by Qi and colleagues, PLIN 6 SNP had been shown to be related to obesity in Malaysian and Indian patients but no correlation was found in Chinese patients [19]. Similarly in our study, we demonstrated PLIN 6 high T-T allele frequency in obese patients. Contrary to our cases, Soenen and colleagues had detected low body weight in patients with homozygote PLIN 6 T-T allele [20].

There are a limited number of studies analyzing PLIN gene polymorphisms and adikopine levels in obesity and leptin was examined in them. We analyzed serum leptin, adiponectin, resistin and ghrelin levels in obese adolescents in the present study and searched their connection with PLIN polymorphisms that are mostly encountered. Leptin was determined significantly high in obese adolescents compared to that of control group. This result is compatible with the common literature data [21]. Nonetheless, other adipokines were established as similar rate in obese and control groups. In literature, there are studies that found adiponectin [22, 23] and ghrelin levels low [24] and resistin levels high in obese patients [25], however, there are also studies that determined similar levels as in our study [26–28]. We detected no correlation between PLIN gene polimorphisms and serum levels of adipokines in our study. Soenen et al. detected significant low levels of leptin in the presence of PLIN 1 C and PLIN 4 A alleles. Low levels of leptin was also detected in females with PLIN 6 T/T allele [20]. Although Meirhaeghe et al. detected no correlation between two most frequent SNPs PLIN 4 (rs894160) and rs4578621 alleles and plasma leptin levels [18].

Superiorities of our study; it is the first research analyzing the relationship of adipokines apart from leptin with perilipins. In addition, it is one of the studies examining perilipin polymorphisms mostly seen in child population and it is the only study performed in Turkish population on this issue. However, relatively low number of patients is the main limitation of this study. Another limitation of the study is the difficulty in comparing our data with the data collected from the same ethnicity due to the lack of the investigation in the subjects.

## Conclusion

We determined PLIN 6 T-T allele frequency high in obese adolescents in our study and found no correlation between any PLIN polymorphism incidence and adipokines. When similar studies were analyzed in the literature, we observe that limited number of studies were concluded alike. These discrepancies may be due to the ethnic factors. Therefore, this conclusion needs to be performed with large number of patient groups and studies in different countries.

## Abbreviations

BMI: Body mass index
BP: Blood pressure
ELISA: Enzyme-linked immunosorbent assay
HDL: High-density lipoprotein
HOMA-IR: Homeostasis model assessment for insulin resistance
LDL: Low-density lipoprotein
PLIN: Perilipin
SNP: Single nucleotide polymorphism
TC: Total cholesterol
TG: Triglyceride
